# Palmistry Notes

**Published:** 1887-11-12

**Authors:** 


### Amusements for Convalescents.

### PALMISTRY NOTES.

By a Lady.

(*Continued from page 58.*)

**Hands, and What They Teach.**

The *philosophic hand* is known by the knotted outline of the fingers, with both joints prominent, and the tips being mixed—some square and others conic. The thumb has the two phalanges of will and logic equally developed, showing a just balance of will and logic. Unconventionality, originality, a power of forming independent judgment on persons and things, reason in all things, admiration of the truly beautiful only, intolerance of tyranny, freedom in social and religious observances, with strong instinctive sense of justice, mark the possessor of the philosophic hand. Among sceptics we find this type of hand largely represented, as the particular bent of mind indicated by the qualities shown above inclines them naturally to treat all matters with freedom. Tolerance, and a capacity for seeing into and looking all round a subject, are also characteristics of the type.

We now turn to the most beautiful, but also to the most impracticable form of hand—the *psychical* or *pointed*—with its delicate, smooth fingers, long and finely pointed, and a slight, sensitive palm. These beautiful hands are rarely met with, and they signify high intuitive gifts and rare refinement of mind. Heart and soul are the inspired source of all their actions, and devotion to beauty in its varied forms the guiding principle of their existence. They have no capacity for leading, although by the fervour of their aspirations they influence greatly those with whom they are brought in contact, and to the less poetical, but more matter-of-fact and practical, square, spatulative hand is reserved the power to embody and utilise the gifts of the psychic subject. When in the exaggeration of the type we find the pointed fingers very accentuated, then there will be the weakness of extremes, and it is with this excess of the type that we get mysticism, credulity, eccentricity, unreliability. Such subjects make the best "mediums," for their smooth fingers offer no opposition to the passage of the electric current. Among Orientals this exaggeration is most frequently met with, and it is productive of spiritualists in abundance.

The *artistic hand* presents three variations of form. In the first we find a supple, well-developed palm, small thumb, and delicate, conical fingers. With this formation of the hand the learner will be governed by inspiration, love of beauty, pleasure, freedom, and variety. In the next variation the hand is large and thick, the thumb big, with the third phalange broad, which denotes luxurious tendencies; and in the third hand, characterised by its unusual size, and its thick, firm palm, self-gratification, love of ease, grandeur, and wealth, will be the principal characteristics. Persons of artistic temperament to whichever of the three variations of the type their hands may belong, are unsuited for the mechanical and physical occupations of life: they are lacking in the stability, foresight, and reflective power which characterise the square and spatulate type. They idealise all things by which they are attracted, and are faithful to their ideal so long only as the glamour with which they have invested it hangs round it. The elasticity and adaptability which characterise the type prevent their taking things very deeply to heart, although they display much fervour and intensity of feeling on occasions when they are moved to excitement; sensitive to the good opinion of others, they shrink instinctively from ridicule. The glow of imagination they mistake for warmth of heart, and under its influence they become generous and impulsive to a fault, and will lavish gifts on their friends, while they treat the demands of their creditors with indifference.

In spite of their failings, when not those of the grosser and luxurious development of the type, persons who are distinguished by the above characteristics will always have many friends and admirers, by reason of their many fascinating qualities, and notwithstanding their unconscious inconstancy and unreliability. There now remains only one more type to consider, and we have done with this department of palmistry.

The *mixed type* stands last, and possesses more than one indication of the various types we have been considering. It is impossible on the present occasion to enter into the numberless variations which are to be found in the mixed hand. Its finger-tips may present the different formations of the square and conic, or conic and spatulate, or psychic and conic; and we may find a long, hollow palm and artistic fingers, and many other variations too numerous and complex to enter into; but I think that what has been said on the indications shown by different formations of the fingers and thumb individually, and of the palm also, on pages 353, 397, 426, will enable those who may wish to delineate a hand of the mixed type to do so without any great difficulty. There is one thing to be noticed when confronted with a mixed hand, that it will be certain to indicate a variety of gifts and characteristics, which will go far to make the owner good at many things, without being greatly gifted in any particular direction, and he will always be an amusing and interesting person, if not very profound or conventional. In my next "notes" I shall take the lines and mounds in the palm for consideration. (*To be continued*).

